# Supplementary material for: Establishing a Proteomics-Based Signature of AKR1C3-Related Genes for Predicting the Prognosis of Prostate Cancer
Source: Int J Mol Sci. 2023 Feb 24;24(5):4513. doi: 10.3390/ijms24054513 (PMC10003753; doi:10.3390/ijms24054513)
Supplement: Supplementary file 1 [file ijms-24-04513-s001.zip › supplementary captions.pdf]

Figure S1: Poor prognosis factor selected by venn.

Figure S2:AKR1C3 associated 18 hub genes Kaplan-Meier plot.

Table S1:The information of each data set.

Table S2: 1164 DEPs were identified in the LNCaP-AKR1C3 group.

Table S3: Summary of the application of proteomics in the diagnosis, progression and prognosis of PCa.

Table S4: The primer sequences.
