# Supplementary material for: Establishing a Proteomics-Based Signature of AKR1C3-Related Genes for Predicting the Prognosis of Prostate Cancer
Source: Int J Mol Sci. 2023 Feb 24;24(5):4513. doi: 10.3390/ijms24054513 (PMC10003753; doi:10.3390/ijms24054513)
Supplement: Supplementary file 1 [file ijms-24-04513-s001.zip › Supplementary Figures.pdf]

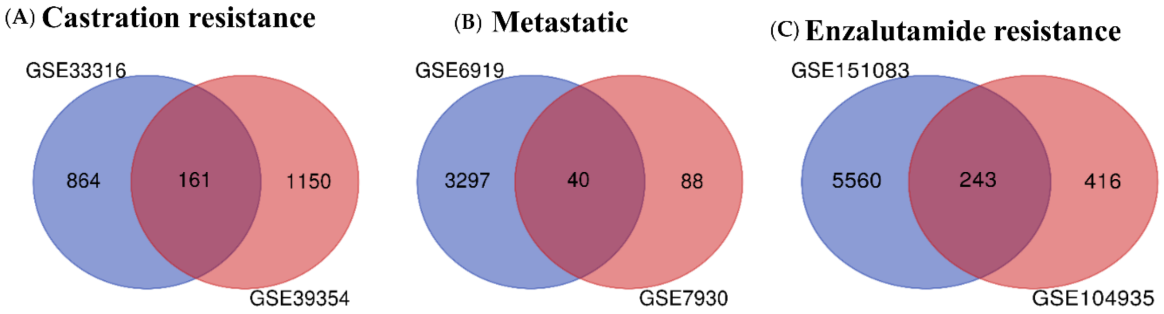

Figure S1. Poor prognosis factor selected by venn (**A**) Castration resistance, (**B**) Metastatic, (**C**) Enzalutamide resistance.

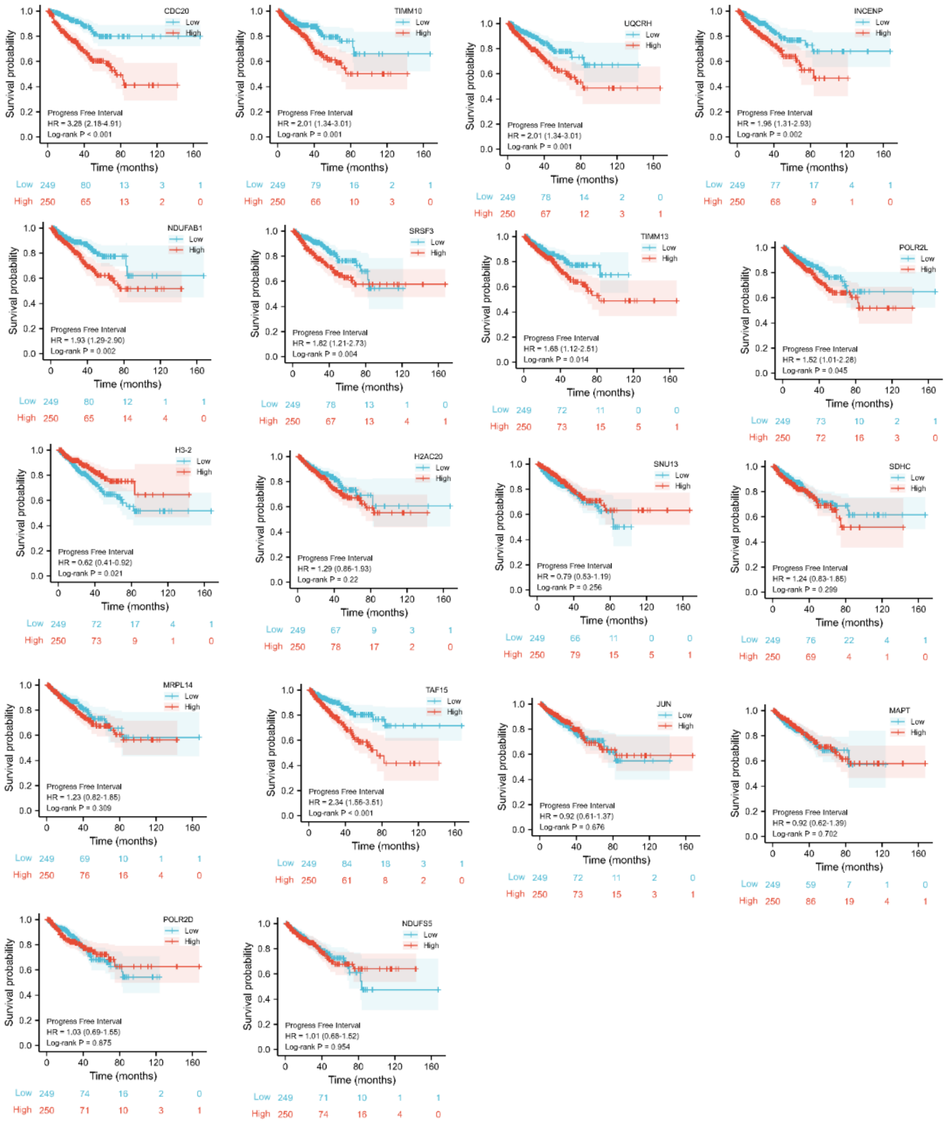

Figure S2. AKR1C3 associated 18 hub genes Kaplan-Meier plot.
