# Supplementary material for: Establishing a Proteomics-Based Signature of AKR1C3-Related Genes for Predicting the Prognosis of Prostate Cancer
Source: Int J Mol Sci. 2023 Feb 24;24(5):4513. doi: 10.3390/ijms24054513 (PMC10003753; doi:10.3390/ijms24054513)
Supplement: Supplementary file 1 [file ijms-24-04513-s001.zip › Supplementary Table S1.pdf]

The information of each data set

Data set	Describe
GSE33316	Compare the gene expression of 5 LuCaP35 xenografts from non-treated mice (Control), and 5 androgen-deprived LuCaP35 xenografts from castrated mice (Castration).LuCaP35-Human prostate xenograft tumor.
GSE39354	The different expressed genes in human prostate VCaP and LNCaP cancer cells. VCaP cells were castration-resistant cells and LNCap cells were not castration-resistant cells.
GES6919	Normal prostate tissue free of any pathological alteration from organ donor and Metastatic prostate tumor samples in para tracheal lymph node from patient
GSE7930	The different genes expressed between metastasis PC3cells and normal cells
GSE151083	C42B and C42B-enzalutamide resistant cells
GSE104935	MDV3100 is an androgen receptor antagonist. This data set shows the difference of gene expression between C4-2B and C4-2B MDV3100 resistant cells
