# Supplementary material for: Establishing a Proteomics-Based Signature of AKR1C3-Related Genes for Predicting the Prognosis of Prostate Cancer
Source: Int J Mol Sci. 2023 Feb 24;24(5):4513. doi: 10.3390/ijms24054513 (PMC10003753; doi:10.3390/ijms24054513)
Supplement: Supplementary file 1 [file ijms-24-04513-s001.zip › Supplementary Table S4.pdf]

The primer sequences.

	forward 5’-3’	reverse 5’-3’
GAPDH	ATGATTCCACCCATGGCA	GATGATGACCCTTTTGGCTC
PSA	CCAAGTTCATGCTGTGTGCT	GGTGTCCTTGATCCACTTCC
FKBP5	GGATATACGCCAACATGTTCAA	CCATTGCTTTATTGGCCTCT
TMRPSS2	CAGGAGTGTACGGGAATGTGATGGT	GATTAGCCGTCTGCCCTCATTTGT
CENPN	TGAACTGACAACAATCCTGAAGG	CTTGCACGCTTTTCCTCACAC
Vimentin	GCCTGCAGGATGAGATTCAGAATA	AACCAGAGGGAGTGAATCCAGATTA
Twist	GCCAGGTACATCGACTTCCTCT	TCCATCCTCCAGACCGAGAAGG
Snail	GGAAGCCTAACTACAGCGAG	CAGAGTCCCAGATGAGCATTG
